# Neuroprotective effects of EDTA on manganese-induced neuroaffective disorders: Implications for anxiety and depressive disorders

**DOI:** 10.1192/j.eurpsy.2025.1020

**Published:** 2025-08-26

**Authors:** H. Harifi, H. Hami, L. Bikjdaouene

**Affiliations:** 1 Laboratory of Biology and Health, Faculty of Science, Ibn Tofail University, Kenitra, Morocco

## Abstract

**Introduction:**

Manganese (Mn), an essential yet potentially toxic metal found naturally in the environment, has been implicated in various neurodegenerative and neuroaffective disorders due to its propensity for chronic intoxication.

**Objectives:**

This study explores the neuroprotective effects of Ethylenediaminetetraacetic acid (EDTA) against Mn-induced toxicity, with a specific focus on its potential to mitigate anxiety and depressive disorders.

**Methods:**

Three groups of male Wistar rats were used: one group was injected with 25 mg/kg Mn, another with 25 mg/kg Mn plus 4 mg/kg EDTA, and the control group received NaCl. Injections were administered intraperitoneally daily for 12 weeks. Rat weights were monitored weekly, and following the exposure period, rats underwent a series of neurobehavioral tests to assess anxiety and depressive behaviors.

**Results:**

Rats receiving the 25 mg/kg Mn dose alone exhibited increased immobility in the forced swimming test (FST) and anhedonia in the sucrose preference test, indicative of depressive behaviors. In contrast, rats treated with Mn and EDTA displayed significantly reduced immobility times and maintained normal sucrose water consumption. Behavioral tests, including the open field test (OFT) and elevated plus maze (EPM), indicated increased anxiety in Mn-exposed rats, which was mitigated by EDTA treatment.

**Conclusions:**

Mn exposure induces anxiety and depressive disorders in rats, but coadministration of EDTA significantly mitigates these effects, suggesting its potential as a neuroprotective agent against Mn toxicity.

**Disclosure of Interest:**

None Declared

